# Revealing Hidden Clues: The “Rubbing Technique” to Enhance Detection of Nodular Basal Cell Carcinoma

**DOI:** 10.1111/srt.70302

**Published:** 2026-04-26

**Authors:** Zyber Hasa, Roberta Vezzoni, Filippo Chersi, Erika Giulioni, Iris Zalaudek

**Affiliations:** ^1^ Dermatology Clinic Maggiore Hospital University of Trieste Trieste Italy; ^2^ Dermatology Clinic Santa Maria degli Angeli Hospital, Friuli‐Venezia Giulia Pordenone Italy

**Keywords:** aesthetic, basal cell, carcinoma, dermoscopy, surgery

## Abstract

Nodular basal cell carcinoma (nBCC) of the face can be challenging to diagnose in its early stages. We report our observation of a simple and rapid clinical maneuver that enhances the visualization of arborizing vessels, erosions, and bleeding, thereby facilitating the identification of nBCC. This preliminary observation suggests that the technique may reduce unnecessary biopsies, preserve cosmetic outcomes, and support timelier patient management.

1

To the Editor,

Nodular basal cell carcinoma (nBCC) commonly affects the head and neck region. Although nBCC generally has a favorable prognosis, lesions located in the so‐called H‐zone of the face may present greater management challenges due to their proximity to critical anatomic structures and potential for local tissue destruction [1]. In these sensitive regions, nBCC can sometimes be challenging to distinguish from other common benign skin lesions, such as dermal nevi or sebaceous hyperplasia, especially in the early stages when diagnostic features are not well‐defined. Biopsy may lead to scarring, so it is important to minimize biopsies in benign cases and enhance diagnostic specificity.

Dermoscopic examination improves diagnostic accuracy by helping to differentiate basal cell carcinomas (BCCs) from other tumors and inflammatory diseases, and early diagnosis may play a key role in reducing both disease‐ and treatment‐related morbidity [2].

Several variants of dermoscopy have been proposed to improve the early diagnosis of BCCs. Ultraviolet‐enhanced fluorescence dermoscopy (UVFD) has shown promise in enhancing lesion visibility, particularly in small, non‐pigmented BCCs. [3] Similarly, super‐high magnification dermoscopy has emerged as a valuable diagnostic tool, providing exceptionally clear visualization of vascular morphology, which may facilitate the detection of characteristic blood vessel patterns in early BCC [4]. However, the use of these techniques is limited by equipment availability and may not be feasible in routine clinical settings.

Simpler clinical maneuvers have also been described. Adler et al. introduced a rubbing technique, in which rubbing a suspected superficial BCC gently with a gauze pad soaked in 70%–80% isopropyl alcohol for 15–30 s enhances the clinical and dermoscopic visualization of erythema, vascular structures, and erosions [5]. This method, though effective, was originally described only for superficial BCC. Building on this, we observed that applying a similar approach to infiltrative lesions such as papules, plaques, or nodules suspected of nBCC, using any transparent skin disinfectant and shortening the rubbing time to 10–20 s, equally enhances key diagnostic features. Arborizing vessels, erosions, and bleeding appear more evident, likely due to localized vasodilation of the tumor's vessels and promotion of erosion through discohesive tumor cells (Figures 1, 2).

**FIGURE 1:**
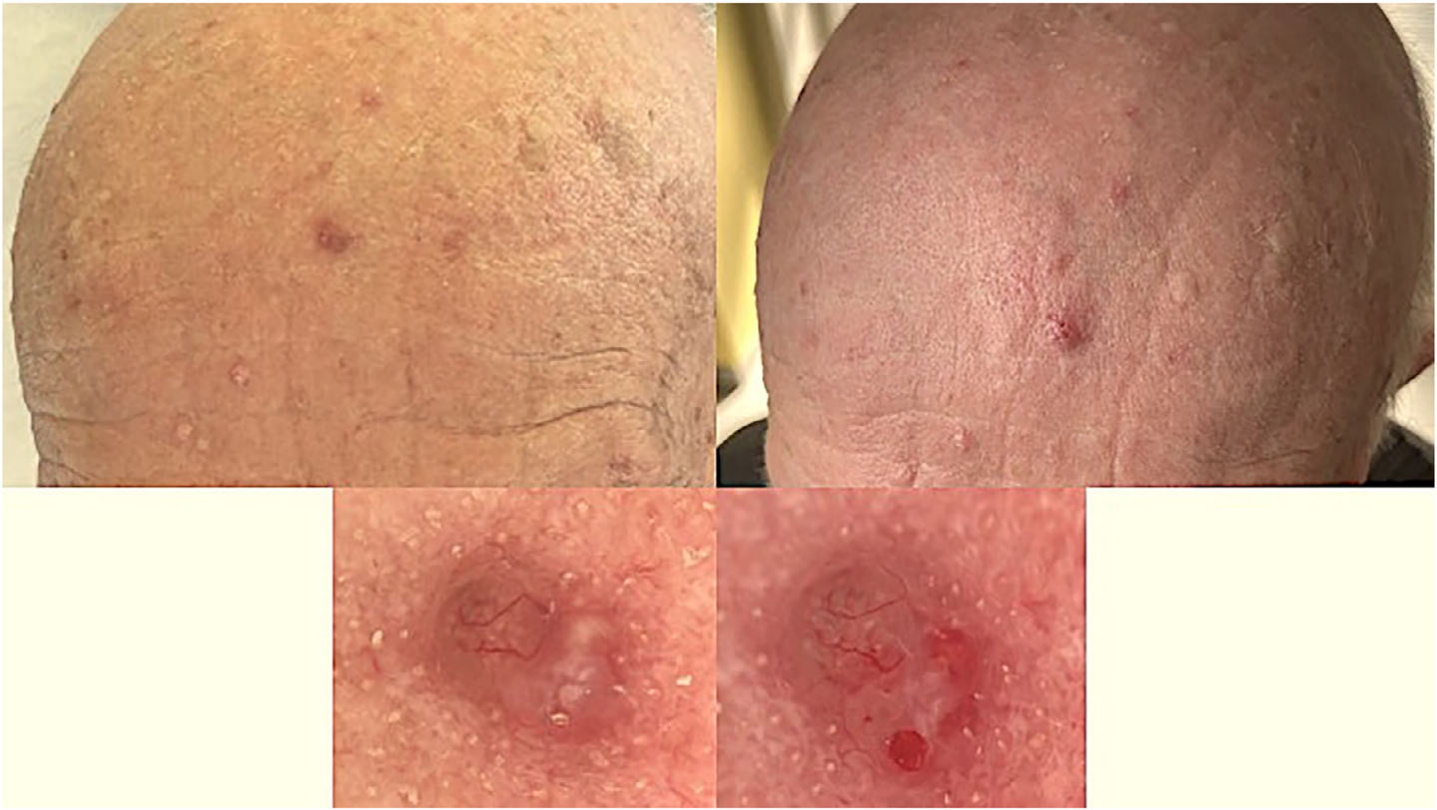
Pre‐ and post‐rubbing images of a histologically confirmed nodular basal cell carcinoma on the forehead of a male patient. The left images show the lesion before, and the right images display the lesion after the rubbing technique highlighting enhanced visibility of arborizing vessels, erosion, and bleeding.

**FIGURE 2:**
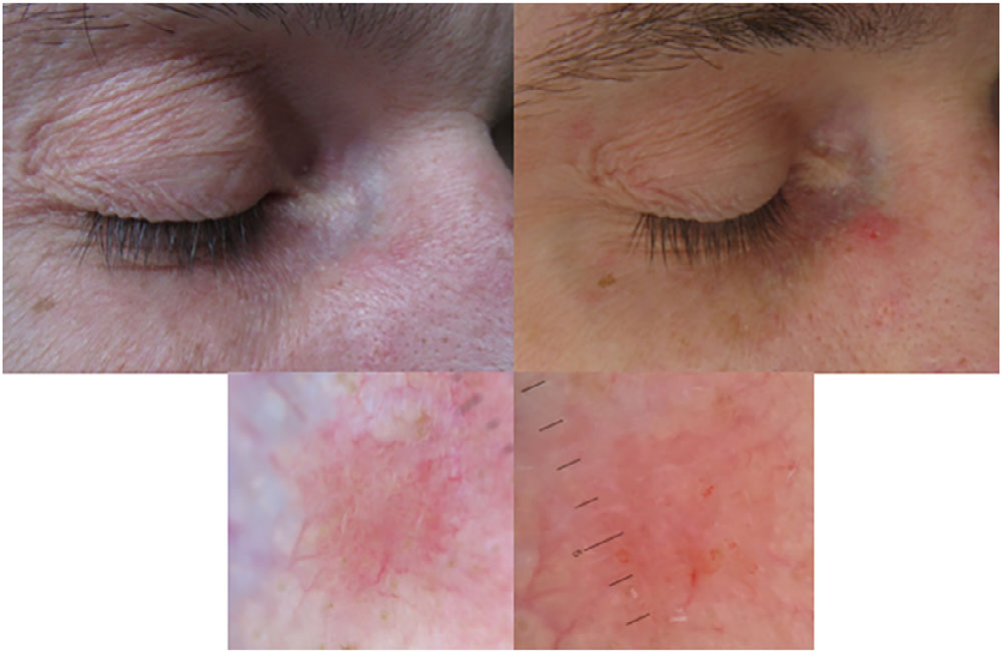
Pre‐ and post‐rubbing images of a histologically confirmed nodular basal cell carcinoma (Pinkus fibroepithelioma) on the right nasal ala of a male patient. The left images show the lesion before, and the right images display the lesion after the rubbing technique highlighting the enhanced erosion, and bleeding.

This technique particularly facilitates the diagnosis of small nBCCs that may not yet display well‐defined dermoscopic features. It may help minimize the extent of surgical excision and scarring. Additionally, it may help more confidently rule out benign conditions that do not exhibit these changes, thereby reducing the need for follow‐up visits or unnecessary procedures.

We propose that incorporating this simple yet clinically effective maneuver into daily practice could improve diagnostic accuracy for nBCC, even among less experienced dermoscopists, optimize treatment timelines, and ultimately improve patient outcomes. This is an exploratory hypothesis that requires validation through controlled clinical studies.

## Funding

The authors received no specific funding for this work.

## Ethics Statement

The patients in this manuscript have given written informed consent to the publication of their case details.

## Conflicts of Interest

The authors declare no conflicts of interest.

## Data Availability

The data that support the findings of this study are available from the corresponding author upon reasonable request.
